# Blood Analytes as Biomarkers of Mechanisms Involved in Alzheimer’s Disease Progression

**DOI:** 10.3390/ijms232113289

**Published:** 2022-10-31

**Authors:** Andrea Baldini, Alberto Greco, Mirko Lomi, Roberta Giannelli, Paola Canale, Andrea Diana, Cristina Dolciotti, Renata Del Carratore, Paolo Bongioanni

**Affiliations:** 1 Department of Information Engineering, Faculty of Engineering, University of Pisa, 56122 Pisa, Italy; 2 Institute of Clinical Physiology, National Research Council, 56124 Pisa, Italy; 3 Department of Biomedical Sciences, University of Cagliari, 09042 Cagliari, Italy; 4 Medical Specialties Department, Azienda Ospedaliero-Universitaria Pisana, 56126 Pisa, Italy; 5 NeuroCare onlus, 56121 Pisa, Italy

**Keywords:** Alzheimer’s disease, blood neurodegenerative biomarkers, generalized linear mixed-effects models, pathways

## Abstract

Alzheimer’s disease (AD) is the leading cause of dementia, but the pathogenetic factors are not yet well known, and the relationships between brain and systemic biochemical derangements and disease onset and progression are unclear. We aim to focus on blood biomarkers for an accurate prognosis of the disease. We used a dataset characterized by longitudinal findings collected over the past 10 years from 90 AD patients. The dataset included 277 observations (both clinical and biochemical ones, encompassing blood analytes encompassing routine profiles for different organs, together with immunoinflammatory and oxidative markers). Subjects were grouped into four severity classes according to the Clinical Dementia Rating (CDR) Scale: mild (CDR = 0.5 and CDR = 1), moderate (CDR = 2), severe (CDR = 3) and very severe (CDR = 4 and CDR = 5). Statistical models were used for the identification of potential blood markers of AD progression. Moreover, we employed the Pathfinder tool of the Reactome database to investigate the biological pathways in which the analytes of interest could be involved. Statistical results reveal an inverse significant relation between four analytes (high-density cholesterol, total cholesterol, iron and ferritin) with AD severity. In addition, the Reactome database suggests that such analytes could be involved in pathways that are altered in AD progression. Indeed, the identified blood markers include molecules that reflect the heterogeneous pathogenetic mechanisms of AD. The combination of such blood analytes might be an early indicator of AD progression and constitute useful therapeutic targets.

## 1. Introduction

Pathogenetic factors of Alzheimer’s disease (AD), the main form of dementia in the world, are not yet known, and the relationships between altered biochemical pathways and disease onset and progression are still unclear. Indeed, currently, a definitive diagnosis of AD and an evaluation of the progression severity can be conducted only after postmortem brain analysis by the assessment of neuropathological markers such as amyloid plaques and neurofibrillary tangles [1]. However, the concurrent observation of gliosis and neuroinflammatory driving cells has highlighted the importance of the mutual relationship between those aberrant proteins and inflammatory molecules often secreted for triggering AD pathological changes [2,3,4,5,6]. In this context, understanding how global perturbations in metabolism and body functions are related to AD neuropathology would be crucial, albeit challenging, for a prompt diagnosis, a correct prognosis and effective treatments [1,7]. A potential tool for AD diagnosis and prognosis is represented by blood biomarkers [8,9,10].

Different technical approaches have been used to look for neurodegeneration-related specific blood biomarkers, such as those of proteomics [11,12] or miRNomics [13,14] or metabolomics [15,16], attempting to detect specific brain molecules —amyloid-beta (Aβ), tau and glial fibrillary acidic protein neurofilaments light chains [17,18]. On the other hand, fewer studies have been performed to assay non-specific blood analytes in patients with neurodegenerative illnesses [19,20], and more specifically AD [11,21,22] to find out novel interesting and useful correlations to brain biomarkers. Levels of blood biomarkers have been recently proposed to predict brain (Aβ) deposition [21] and, consequently, a cognitive impairment. In our study, we aimed to correlate patients’ routine blood analytes, often requested by general practitioners, to AD progression by using a rigorous and robust statistical modeling approach.

From a methodological point of view, in previous studies, the computational evaluation of AD severity indexes and biomarkers often relies on basic statistics [23] and machine learning techniques [24]. However, the clinical datasets are often characterized by repeated (dependent) measures and non-Gaussian categorical indexes, such as those indicating the severity of the disease. These critical issues are often addressed by transforming the categorical data, using non-parametric tests, or relying on classical ANOVA robustness to non-normality. Nevertheless, the application of such strategies could violate many required assumptions of statistical tests and models (e.g., independence of data) or reduce the quantity of data (averaging repeated measures), limiting the robustness of results and hiding possible remarkable findings. On the other hand, machine learning methods afford an easy-to-use tool to predict categorical variables but often represent “black box” models and it is not straightforward to interpret how the input variables are combined to make predictions.

In this study, we built a database including longitudinal observation of several routine blood analytes of AD patients with different levels of disease severity. The blood analytes were used to build mathematical models to investigate their possible role in inferring the state of AD progression. In this scenario, to overcome the aforementioned methodological limitations, we applied generalized linear mixed-effects models accounting for repeated measures and hierarchical data structures. In addition, we investigated whether the identified potential disease-severity-related analytes converge towards common pathways of neurodegeneration. The correlation of pathways with AD is discussed.

## 2. Materials and Methods

### 2.1. Patient Population and Clinical Data

#### 2.1.1. Study Population

The dataset was characterized by longitudinal observations collected over the past 10 years (from 2012 to 2022) from 90 late-onset AD patients (54 women; age 84 ± 4.5). The dataset included 277 observations, each of which was characterized by both clinical and biochemical information. Particularly, each observation combined the Clinical Dementia Rating (CDR) score [25] and the values of 61 blood analytes describing routine profiles of kidney, liver, pancreas and heart functions, parameters of hemostasis and metabolism and markers of inflammatory, oxidative and immunological activity (see Table 1).

The clinical information is summarized by the CDR, which represents a global dementia staging instrument primarily conceived for use in persons with dementia of the Alzheimer’s type. The CDR score is derived from a standard set of information that encapsulates semistructured outcomes from other well-known scales [26] and cognitive tests [27]. Of note, the CDR rates the patient’s cognitive performance in six different domains that are usually assessed with separate cognitive tests: memory, orientation, judgment and problem solving, community affairs, home and hobbies and personal care. The global CDR is derived from a synthesis of the individual domains’ ratings, and it is expressed on six levels: 0.5 (questionable), 1 (mild), 2 (moderate), 3 (severe), 4 (very severe) and 5 (extremely severe).

In addition, data regarding comorbidities were collected alongside AD-specific clinical information. In particular, among the study population, 26% of the patients suffered from dyslipidemias and were treated using statins, 40% from cardiovascular diseases or hypertension, 15% were affected by diabetes Mellitus and 18% suffered from dysthyroidism. Additionally, half of the patients suffered from depression. Each comorbidity was regularly diagnosed after a medical evaluation.

Twenty-seven healthy subjects (15 females; age 80 ± 4.5) were also recruited as the control population, and their blood samples were acquired over time. Each control subject was classified as such, following the same clinical assessment protocol as AD patients.

All blood samples were withdrawn in the mornings (between 8.00 a.m. to 9.30 a.m.) from fasting patients and were analyzed according to the good laboratory practice (GLP) protocol by the laboratory of chemical–biochemical analysis of the Azienda Ospedaliera Universitaria di Pisa (AOUP).

The number of blood withdrawals per subject varied from a minimum of one to a maximum of thirteen.

#### 2.1.2. Inclusion and Exclusion Criteria

The following inclusion and exclusion criteria for patients’ enrollment were established. Subjects of both sexes, aged from 60 to 90 years, were considered for the study. We recruited subjects with premorbid conditions (i.e., Mild Cognitive Impairment (MCI) with both single and multiple cognitive domain impairment according to the diagnostic criteria of Petersen et al. [28]) and patients with a diagnosis of Alzheimer’s Disease (AD) with severe cognitive impairment according to the NINDS-ARDA criteria [29].

We excluded from the study patients with a diagnosis of subcortical dementia (such as Lewy Body Disease Dementia [30] or other neurodegenerative diseases) and demented patients with a diagnosis of vascular dementia according to the NINDS- AIREN criteria [31,32] or with a prevalent vascular component (i.e., “mixed dementia” [33], characterized by the coexistence of degenerative and cerebrovascular disease in the same demented patient). In addition, patients with severe psychiatric disorders, traumatic brain injury, pseudo-dementia or neoplastic disease in the last 5 years were not eligible for the study.

#### 2.1.3. Screening Procedures

According to international guidelines of good clinical practice (Vayamanthan et al. 2008), the following Protocol was applied. In order to verify enrollment criteria, data regarding the clinical history and pharmacologic treatments for the cognitive deficit and possible comorbidities were collected. All subjects performed a battery of neuropsychological tests, including the Mini-Mental State Examination (MMSE) [34], the Montreal Cognitive Assessment (MoCA) [35] and the Milan Overall Dementia Assessment (MODA) [36], which were used—together with the Activity Daily Living (ADL) and Instrumental Activity Daily Living (IADL) scales [37]—to obtain clinical data on both cognition and functional autonomy to assign the correct Clinical Dementia Rating (CDR) [25] scores. Furthermore, we employed the Beck Depression Inventory (BDI) [38] for mood disorders. The modified Hachinski Ischemic Scale (HIS) [39] was used for differentiating clinical types of dementia (primary degenerative, vascular or multi-infarct and mixed type). Moreover, all subjects performed brain Magnetic Resonance Imaging (MRI) in order to support the differential diagnoses, between degenerative and vascular dementia, according to specific cortical and subcortical changes having diagnostic significance. Particularly, reduced cortical thickness, enhanced perivascular spaces and impairment of hippocampus volume, all remarkable signs of degenerative dementia, and focal ischemic lesions as hallmarks of vascular dementia, were considered [40]. Only for a few patients, further confirmation of AD diagnosis correctness was given by cerebrospinal fluid (CSF) amyloid and tau biomarkers analyses. Of note, magnetic resonance and biomarkers tests were not repeated over time during the follow-up evaluations due to their invasiveness or economic cost. Finally, the subjects with a suspected diagnosis of dementia performed brain ${}^{18}$F-deoxyglucose (FDG)-Positron emission tomography (PET), which provides as an early pre-clinical biomarker a pattern of reduced brain FDG metabolism [41].

### 2.2. Dataset Manipulation

Each patient was followed over time, and his/her blood data were collected about every three months until their death or when they stopped their periodic visits. Consequently, (i) not all patients had the same number of observations over time, and (ii) each CDR level was associated with a different number of samples. According to the study’s aim of investigating AD progression both at a single-subject and group level, we considered in the analysis only those patients with at least four blood samples collected over time. In this way, we mitigated the risk of fitting models at the single-subject level with an insufficient number of points. As a result of this manipulation, we performed the statistical analysis considering eighteen patients with a number of blood samples from 4 to 5, thirteen acquired from 5 to 7 times, and 3 with more than 7 withdrawals. To define the different severity levels, we built on the CDR scale. In more detail, for the sake of clarity and to reduce the risk of overfitting, we considered 4 levels of AD severity, grouping the CDR scores as follows: (i) mild (CDR = 0.5 or CDR = 1), moderate (CDR = 2), severe (CDR = 3) and very severe (CDR = 4 or CDR = 5). Accordingly, 45 patients’ blood samples were labeled as mildly severe, 44 as moderately severe, 50 as severe and 47 as very severe. The statistical modeling, described in the next section, was performed by modeling these aggregated classes.

### 2.3. Model Definition

We designed computational models aimed at explaining AD severity, described through the CDR score, as a function of the measured blood analytes. The four levels of severity (mild, moderate, severe and very severe) were considered as an ordinal categorical variable. To model the trajectory of such a non-Gaussian variable without neglecting the data interdependency depending on the subjects’ repeated measures, we adopted a mixed-effects modeling approach. More specifically, we took advantage of the ordered logit mixed-effects (OLME) framework, which accounts for binomial distributed residuals using a logit link function. Accordingly, each analyte in the dataset was used as a fixed effect in an OLME model of AD severity, including a subject-varying random intercept that accounted for the data’s idiosyncrasies. The general expression for the models is formally defined in Equation (1):

(1) $$\left\{\begin{array}{c} Pr\left(Y_{ij}|a_{i}>k\right)={\mathrm{logit}}^{-1}\left(a_{i}+x_{ij}\cdot \beta -\theta_{k}\right)\\ a_{i}∼N\left(0,\sigma^{2}\right)\\ \mathrm{logit}\left(p\right)=\ln\left(\frac{p}{1-p}\right) \end{array}\right.$$

where $Y_{ij}$ represents the *j*th severity level collected from the *i*th subject, *k* defines the *k*th-ordered class of AD severity, $a_{i}$ indicates the normally distributed random intercept with zero mean and $\sigma_{a}^{2}$ variance, $x_{ij}$ is the analyte measured from the *i*th subject during the *j*th acquisition and $\theta_{k}$ can be interpreted as a threshold parameter characterizing the *k*th class of AD severity.

### 2.4. Model Fitting

**Figure 1:**
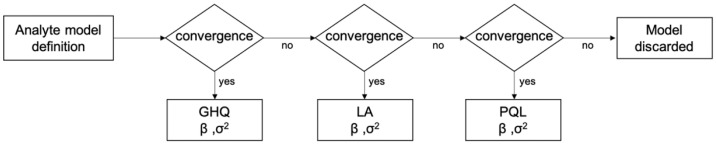
OLME model parameters estimation procedure.

To identify the parameters of the models, we estimated the maximum likelihood (ML) of both the blood analyte fixed effect (β) and the standard deviation of the random intercept ($\sigma_{a}$). In a hierarchical context, the ML parameters’ estimation requires integrating likelihoods over all possible values of the random effects. To this aim, multiple strategies can be implemented, characterized by different accuracy and speed of convergence. They include the penalized quasi-likelihood (PQL) [42], the Laplace approximation (LA) [43] and the Gauss–Hermite Quadrature (GHQ) [44]. Given its proven higher estimation accuracy [45], we adopted a GHQ over 20 quadrature points for the analyte OLME model fitting. Whenever the model related to a specific analyte failed to converge with such a GHQ method, it was re-fitted using the LA approach. Finally, in the very few cases where the model did not converge with either the GHQ or LA, the PQL method was used. The analytes models, which due to their non-linear nature did not converge with any of these 3 methods, were excluded from the analysis. The fitting procedure is summarized in Figure 1.

### 2.5. Statistical Inference

We performed two statistical analyses: (i) the statistical significance of the analytes was evaluated using the Wald Z test [45] under the null hypothesis of no effects of the models’ analytes. Additionally, we estimated the contribution of each significant analyte in terms of explained variance by computing both the conditional and marginal $R^{2}$[46]. In particular, the marginal $R^{2}$ defines the variance explained by the only fixed effects, while the conditional $R^{2}$ indicates the variance explained by the whole model including the random effects. The significant models were ranked based on their *p*-values. (ii) A statistical comparison between the control population and the AD patients at each severity level (i.e., mild AD, moderate AD, severe AD and very severe AD) was performed for each analyte using a non-parametric Mann–Whitney test under the null hypothesis of no difference between AD patients and control subjects. Repeated measures were averaged to consider an unpaired data design, and the Bonferroni correction was applied to compensate for multiple comparisons.

### 2.6. Blood Analytes Examination

Blood analytes that resulted statistically significantly related to AD severity were examined through the Reactome pathway databases, an online bioinformatics tool supplying integrated analyses of the biologic reaction network, which can be used to search how analytes perform their function through specific pathways [47]. More specifically, the Reactome database is an open-source knowledge base of biomolecular pathways providing tools for basic research, genome analysis, modeling and systems biology. Using the aforementioned database, an over-representation analysis was performed to detect the pathways in which the deferentially expressed biomolecules exceed the number that could be randomly expected. In Reactome, the statistical significance of each pathway was calculated through the combination of the Binomial Test and the False Discovery Rate (FDR) correction to avoid misleading results. The FDR correction was applied using the Benjamini–Hochberg approach.

## 3. Results

Supplementary Table S1 reports descriptive statistics, including the grand mean and the corresponding severity class mean and standard deviation for each analyte reported in Table S1.

### 3.1. Statistical Modeling Evaluation

Each model converged with at least one of the three fitting methods. Thus, no analytes were excluded from the analysis. The Wald Z test revealed a total of eight statistically significant analytes. The estimated significant β parameter and the results from the statistical analysis, including marginal and conditional $R^{2}$, are reported in Table 2.

Among the significant analytes, three of them resulted significantly positively correlated with AD progression, while an inverse relationship with the severity index was observed for six of them. Particularly, creatinine (p=0.0015, β=7.403), Na (p=0.0345, β=0.282) and Cl (p=0.0025, β=0.313) increased as the severity of the disease grew. Conversely, HDL-cholesterol (p=0.0013, β=−0.157), Ferritin (p=0.0090, β=−0.017), Fe (p=0.0154, β=−0.031), total cholesterol (p=0.0241, β=−0.031) and LDH (p=0.0430, β=−0.022) tended to decrease for the higher values of severity level. More precisely, in this ordered multinomial context, the slopes of the regression variables (i.e., the β parameters) do not indicate a linear increasing or decreasing trend as in a standard correlation analysis. Indeed, they correspond to the dependent variable’s change in the log-odds scale resulting from a one-unit variation of the regressor in its proper measurement scale. It is worthwhile noting that the negatively correlated ones were the most informative (i.e., higher values of $R^{2}$), with a unique exception represented by creatinine, explaining a relevant 16.9% of AD severity variability.

A detailed description of the statistical results of the comparison between controls and the patients with different severity levels can be found in Supplementary Table S2 Only a small percentage of analytes significantly differed from the control values, regardless of the severity class of the observations. It is worthwhile noting that among the analytes significantly related to AD progression, only chlorine showed a significant difference between the control group and the AD patient with mild severity (*p* = 0.002).

### 3.2. Biomarker Analysis and Pathway Search

The role of each analyte that resulted significantly related with AD severity was examined through the Reactome databases to identify the pathways linked to their differential expression. A detailed description of the pathways involved is reported in Table 3. Results show the convergence towards *Immune system*, *Transport of small molecules* and *Vescicle-mediated transport*.

Of note, among the significantly AD severity-related analytes, only Na, Cl, total and HDL-cholesterol, Fe and Ferritin were involved in the identified pathways. Particularly, Ferritin, Fe, Total cholesterol and HDL-cholesterol were implicated in vesicle-mediated transport. Fe and Ferritin were also involved in the transport of small molecules. Moreover, we observed a significant role of six significant analytes in the network of biological processes implicated in the immune system (i.e., Ferritin, Fe, Total cholesterol, Cl and Na). Although statistically significant effects on CDR progression of LDH and creatinine were observed, no resulting pathways included these molecules.

## 4. Discussion

In this study, we performed a statistical model analysis to identify blood biomarkers significantly associated with AD progression. To address all the critical issues related to the hierarchical structure of the dataset without neglecting the repeated measures over time, we mathematically modeled diachronic variations of subject-dependent dynamics. Specifically, we adopted generalized linear mixed-effects models to account for random variability among subjects and fully match the nature of the AD severity index, providing a powerful tool to make robust inferences of regressors (i.e., no violated statistical assumptions of the model) and easily interpret their contribution to the model. As a result, the correlation between CDR and specific blood analytes was thoroughly quantified.

From the generalized linear mixed-effects model, eight analytes resulted significantly related to AD severity. Among these, due to their very weak relationship with AD severity ($R^{2}$ = 0.002), Na and Cl are not further discussed. On the other hand, Fe, ferritin and total and HDL-cholesterol were all inversely related to AD severity and were shown by the Reactome database analysis to be involved in the subpathway of small molecules and vesicle-mediated transport and to converge towards an overactivation of scavenger receptors (SRs). SRs consist of a broad family of multifunctional proteins found on the membrane of a variety of cells, including microglial cells, involved in the binding and clearance of toxic ligands [48,49]. This altered signal could be indicative of an increased molecule trafficking during the neuroinflammation progression [50,51,52]. Moreover, different SRs are expressed both by endothelial cells and by a plethora of leukocytes such as lymphocytes and neutrophils [53,54]. Therefore, these immune responsive cells coming from systemic circulation can undesirably sustain and exacerbate inflammation already taking place in AD-affected brains by the recruitment of resident glial and macrophage cells. In this way, peripheral leukocytes could infiltrate the brain by crossing the brain–blood barrier (BBB) either in normal conditions or, more dramatically, as a result of the damage of the same barrier, often invoked but not definitely assessed to play a boosting effect in the progression of AD. SRs share the ability to recognize and internalize modified low-density lipoproteins (LDL) and several other ligands, including Aβ, in both its fibrillary and soluble form, suggesting the fascinating interpretation of an alternative compensatory mechanism for Aβ clearance [55].

One of the main unresolved questions in neurodegenerative diseases is whether neuroinflammation stands after aberrant protein accumulations or if it is causative of their production. Aβ plaques have been considered for many years as the main cause of neurotoxicity in AD onset and progression, but development of Aβ plaques is a common phenomenon in most people during aging and does not necessarily lead to cognitive decline. As a matter of fact, based on postmortem analyses, individuals with Aβ plaques and large Fe deposits are highly likely to develop dementia [56], possibly implicating that a tendency towards brain Fe accumulation could be a factor increasing AD susceptibility. In addition, during the onset of AD, microglial cells are believed to protect the brain by incorporating extracellular Aβ filaments that are prone to bind several metals, such as Cu, Zn and Fe [49,57], until their maximum buffering capacity [57]. In this way, across years, Fe ions could increase and accumulate within the brain.

In humans, Fe is incorporated into proteins as a component of heme and is involved in numerous oxidation-reduction reactions, such as those involved in erythropoiesis, energy production, lipid peroxidation, myelin formation, neurotransmitters’ synthesis and cellular immune responses. Circulating and cellular Fe is linked to proteins and other transporters, such as ferritin, to secure its vital functions and limit its potential toxicity. Fe can enter the cell via transferrin 1 receptor and be reduced from ferric to ferrous form via a metalloreductase in the endosome: in such a form, Fe can be stored in ferritin, or exported from the cell through ferroportin [56,58]. Fe accumulation with age in several brain compartments has been known for a long time, even though no satisfactory explanation has been drawn on that regard, including all the related consequences and implications [49]. Since brain Fe content increases more dramatically during neurodegenerative diseases, causing the onset of sideroptosis (ferroptosis), an Fe-dependent and lipid peroxidation-driven type of programmed cell death [59], it is likely that ferroptosis may offer an additional contribution to neurodegeneration in AD. Ferritin degradation through the nuclear receptor coactivator 4 (NCOA4) contributes to ferroptosis by increasing the free intracellular Fe levels [58]. Dysregulation of NCOA4-mediated ferritinophagy disrupts systemic Fe homeostasis with deleterious effects on oxidative stress modulation, leading ultimately to enhanced neurodegeneration [60]. Fe accumulation [61], lipid peroxidation [58] and mitochondrial dysfunction [62], the main hallmarks of ferroptosis, are observed early in AD pathology, suggesting that targeting ferroptosis in AD may lead to the prevention of symptoms’ manifestation such as cognitive decline. Given the dysregulation of intraneuronal Fe homeostasis, more and more blood Fe from the labile pool is consumed and, on the other hand, brain ferritin molecules are numerically less sufficient for Fe storage, which tends to accumulate in excess in neurons. Our findings regarding a progressive reduction in plasma Fe and ferritin concentrations in AD patients in respect to disease severity worsening might correlate with increased demand for Fe and ferritin by the brain.

The possibility to track ferritin and Fe in blood during the first neurodegenerative signals might offer a potential early marker for AD and point out possible therapeutic targets [58,63,64,65]. Other molecules we found to be reduced in blood inversely to AD severity are total cholesterol and HDL-cholesterol. Cholesterol amounts measured in blood include HDL- and LDL-cholesterol, intermediate density lipoprotein (IDL), very-low-density lipoprotein (VLDL)-cholesterol and oxysterols. Cholesterol constitutes the main building block for brain development, accounting for about 23–25% of total body cholesterol as the main component of cellular membranes, therefore involved in enriching the myelin sheath, in signal conduction and in synaptic vesicles production for neurotransmission function [66]. A large portion of the cholesterol pool is synthesized directly inside the brain (namely by astrocytes and neurons), whereas a small portion is exchanged between the brain and blood circulation in the form of oxysterol metabolites, such as 24S-hydroxycholesterol (24S-OHC) and 27-hydroxycholesterol (27-OHC). Although the paramount role of for normal brain metabolism, its specific significance in the pathogenesis of neurodegenerative diseases has not been fully elucidated.

Many studies report conflicting results related to the risk of dementia in people with high blood cholesterol [67] or low HDL-cholesterol [68] and on the balance between brain and blood cholesterol amounts [69]. In healthy people, the BBB prevents the passage of cholesterol from the blood to the brain but, at the onset of neuroinflammation, the BBB undergoes a destructive process caused indeed by inflammatory mediators such as reactive oxygen species (ROS), neutrophils-released metalloproteases and Fenton reaction [70]. Cholesterol has been shown to accumulate in mature Aβ-plaques in AD patients [69], and its brain levels positively correlate with the disease severity in AD patients. Cholesterol balance is finely regulated, but the causes of its accumulation in the brain remain still unclear [24]. In healthy conditions, excess of cholesterol in the brain is converted into 24S-OHC (by cytochrome Cyp46A1) to make it suitable for diffusion across the BBB from the brain. Remarkably, almost the total amount of plasmatic 24S-OHC is derived from brain metabolism, making this latter an appealing biomarker to monitor cholesterol turnover and fluctuations in the brain related to different stages of AD [71]. Recent papers have investigated the displacements of cholesterol between the brain and peripheral blood during various diseases, including neurodegenerative, however, its role remains still controversial [50,51,52]. Indeed, it has been already shown that 24S-OHC is elevated in early or mild AD but decreased in more advanced illness stages [71].

27-OHC is a cholesterol metabolite that, after having been synthetized in various cellular compartments of the body, is able to enter through the BBB and, once into the brain, be converted into 7A-hydroxy-3-oxo-4-cholestenoic acid (7HOCA) by Cyp7B1 [72]. Notably, upon neuroinflammatory conditions, 27-OHC uptake is increased, likewise, to restore demyelination or microglia activation. Moreover, since the activities of Cyp46A1 and Cyp7B1 are suppressed, the efflux into the blood is reduced, whereas retention in the brain increases [72]. Another explanation for enhanced cholesterol and Fe brain levels might be the occurrence of hemolytic events fostered by the increased oxidation milieu in AD brains; in fact, enhanced ROS amounts in the brain consequent to microglia activation together with Fe overload may cause hemolysis that, in turn, might cause further release of Fe and cholesterol [73]. This could provide a reasonable explanation for the total cholesterol progressive reduction in blood along the AD progression [74]. It is known that 27-OHC is the most abundant oxysterol in the circulation and HDL-cholesterol is its main carrier in plasma [75]. Changes in the concentrations of HDL-cholesterol are paralleled by equidirectional changes in HDL-27-OHC levels, thus indicating that HDL acts as a passive acceptor and transporter of 27-OHC, explaining up to 40% of the variation in HDL-27-OHC levels. Our findings regarding a progressive reduction in HDL-cholesterols in plasma from AD patients according to their disease worsening might correlate with the alteration in cholesterol turnover in the brain. Therefore, dysregulation of cholesterol transport and Fe metabolism in the central nervous system contributes to poor prognoses of AD.

Notably, the comparison between a control group and the AD patients showed that the potential blood biomarkers of AD progression did not significantly differ from the healthy interval. This suggests that the relevant information of such analytes is not in the value itself but in their dynamic trend as the severity of the disease varies. Although some of the analyte values could be altered by the drugs to correct or mitigate comorbidities (e.g., statins), our statistical results are not significantly affected by this. Indeed, our mathematical framework intrinsically models intrasubject variations of CDR as a function of blood analytes. Thus, since the patients were treated in the same way over periodic medical visits, the drug’s effect should not affect the model coefficient, which describes the relationship between the analytes and AD severity, but only the model intercept. This aspect makes our mathematically rigorous models even more crucial and relevant. Indeed, the values of such analytes, within the healthy range, either naturally or recovered through the use of specific drugs, could hide the potential informative power of the identified analytes to the common clinical practice often based on the clinician’s experience.

A possible limitation of the study is the fact that the investigated blood analytes are not AD-specific parameters. This, alongside the fact that the models adopted do not allow causality considerations, could lead to the risk of an overspeculation. However, although limited in number, there are already other studies in the literature that have shown robust and interesting results about the relationship between routine blood analytes and AD [6,21], supporting their possible role as AD biomarkers. It should also be considered that the investigation of non-specific parameters could have a great impact on the study of AD, in fact, contrary to the most common specific biomarkers (e.g., CSF, tau), these are much less invasive, easier to be collected, and less expensive. In this regard, a comparison with AD-specific biomarkers would have been interesting but not possible. Indeed, patients would have undergone an invasive procedure (e.g., lumbar punctures) during every follow-up visit (approximately every three months). Thus, contrary to blood withdrawals representing an easy-to-sample tool, it was not possible to include these procedures so frequently in the common clinical routine.

## 5. Conclusions

Our results show that AD progression significantly are correlated with levels of Fe, ferritin and cholesterol, which might be considered as markers of the progression of cognitive impairment. The inverse relationship with AD progression can be due to their higher brain retention already at the onset of the cognitive impairment, although further research work is needed. Indeed, an initial increase in Fe, ferritin and cholesterol, together with Aβ plaques, could saturate the microglia and also some neurons, causing an accumulation of these substances, with consequent loss of functions up to cell death. Such events might lead to the recall of these analytes from the blood with consequent progressive reductions in plasma Fe, ferritin and cholesterol levels; the more evident, the more the disease progresses.

This work paves the path towards an objective and rigorous support system that could help clinicians to an early prediction of the course of the disease and could lead to innovative targets in host-directed therapies. Moreover, it gives new insight that could support the research of an early diagnosis of AD. Future studies will investigate the relationship between non-specific blood analytes and the progression of other forms of dementia (e.g., Fronto-temporal dementia, Lewy body dementia) as well as other neurodegenerative diseases often associated with dementia symptoms (e.g., Parkinson disease [76]). Our methodological approach could highlight similarities and differences with AD, providing not only a tool for a more objective prognosis of such different forms of dementia but also a powerful approach for a differential diagnosis. To this aim, we will focus not only on increasing the number of patients in the dataset, opening the recruitment to other forms of dementia, but we will also expand the analytes set, also introducing synthetic variables derived from a linear or non-linear combination of blood analytes, as in [76].

## Figures and Tables

**Table 1:** List of Analytes used in the study.

Inflammation and Immunology		Plt	Platelets
Bas	Absolute basophils count	PT	Protrombin time
Bas%	Basophils percentage	PTT	Partial thromboplastin time
Eos	Absolute eosinophils count	PTTr	Partial thromboplastin time ratio
Eos%	Eosinophils percentage	RBC	Red blood cells
ESR	Erytrocyte sedimentation rate	**Metabolism**	
Fibr	Fibrinogen		
gGl	Gamma globulins	ALT	Alanine aminotransferase
Lym	Absolute lymphocytes count	Amy	Amylase
Lym%	Lymphocytes percentage	AST	Aspartate aminotransferase
Mon	Monocytes absolute count	BA	Biliary acids
Mon%	Monocytes percentage	Bil	Bilirubin
Neu	Neutrophils absolute count	Chol	Total cholesterol
Neu%	Neutrophils percentage	CK	Creatine kinase
PAlb	Prealbumin	Cre	Creatinine
WBC	White blood cells	Fe	Iron
**Basics**		Fer	Ferritin
a-1Gl	Alpha-1 globulins	FA	Folic acid
a-2Gl	Alpha-2 globulins	gGT	Gamma glutamyltransferase
Alb	Albumin	Glu	Glucose
βGl	Beta globulins	HDL-Chol	High density lipoprotein cholesterol
Ca	Calcium	LA	Lactic acid
Cl	Chloride	LDH	Lactic dehydrogenase
Hb	Hemoglobin	Lip	Lipase
Hct	Hematocrit	Trig	Triglicerids
INR	International normalized ratio	Urea	Urea
K	Potassium	VitB12	Vitamin B12
MCH	Mean corpuscule hemoglobin	**Oxidative Stress**	
MCHC	Mean corpuscule hemoglobin content	FRD	Free radical derivatives
MCV	Mean corpuscule volume	GPx	Glutathione peroxidase
Mg	Magnesium	GR	Glutathione reductase
Na	Sodium	SOD	Superoxide dismutase
P	Phosphorus	TPAO	Total plasma antioxidants

**Table 2:** Results of the statistical evaluation. “*p*” column contains, for each analyte, the result of the Wald test in terms of *p*-value. The “β” column shows the slope of the corresponding model. The “$R^{2}$” column reports the CDR variability explained by the only model’s fixed effect, and the last column provides the fitting method for each analyte model.

Blood Analyte	*p*	β	$R^{2}$	Fitting Method
HDL-cholesterol	0.0013	−0.157	0.197	GHQ
Creatinine	0.0015	7.403	0.169	GHQ
Cl	0.0025	0.313	0.002	PQL
Ferritin	0.0090	−0.017	0.155	GHQ
Fe	0.0154	−0.031	0.051	GHQ
Total cholesterol	0.0241	−0.031	0.046	GHQ
Na	0.0345	0.282	0.002	PQL
LDH	0.0430	−0.022	0.032	GHQ

**Table 3:** List of the main significantpathways identified by the analysis on the Reactome database (Column 2); Student’s *t*-test: p<0.01 (FDR).

Pathway	Identified Analytes	*p*-Value	Involved Subpathways
Transport of small molecules	Ferritin, Fe	1.65 × 10${}^{-2}$	Fe uptake and transport
Vesicle-mediated transport	Ferritin, Fe, total cholesterol, HDL-cholesterol	1.59 × 10${}^{-2}$	Fe uptake and transport, Scavenging by Class A Receptors, Binding and uptake of ligands by Scavenger Receptors, Membrane trafficking
Innate immune system	Ferritin, Fe, total cholesterol, Cl, Na	1.78 × 10${}^{-2}$	Scavenging by Class A Receptors

## Data Availability

The data presented in this study are available on request from the corresponding author.

## Supplementary Materials

The following supporting information can be downloaded at: https://www.mdpi.com/article/10.3390/ijms232113289/s1.
