# Supplementary material for: Blood Analytes as Biomarkers of Mechanisms Involved in Alzheimer’s Disease Progression
Source: Int J Mol Sci. 2022 Oct 31;23(21):13289. doi: 10.3390/ijms232113289 (PMC9657599; doi:10.3390/ijms232113289)
Supplement: Supplementary file 1 [file ijms-23-13289-s001.zip › ijms-1924266-supplementary.pdf]

Supplementary Table S1: Analyte=analyte acronyms; n=number of patients;Mild=mean and standard deviation of mild class (CDR=0 U CDR=1); Moderate=mean and standard deviation of moderate class (CDR=2); Severe=mean and standard deviation of severe class (CDR=3); Very severe=mean and standard deviation of very severe class (CDR=4 U CDR=5).

Analyte	n	Mild	Moderate	Severe	Very severe
Glu (mg/dL)	30	99.91±24.58	99.26±23.90	99.91±33.06	90.04±21.08
Urea (mg/dL)	30	39.43±10.93	42.95±14.70	42.68±16.42	41.86±15.87
Cre (mg/dL)	30	0.82±0.18	2.88±13.73	1.04±0.28	0.88±0.24
LA (mg/mL)	30	12.57±3.97	14.25±12.86	14.15±7.55	14.67±5.99
Bil (mg/dL)	30	0.56±0.25	2.51±13.79	0.47±0.16	0.49±0.21
BA (µmol/L)	30	9.95±5.32	9.72±12.89	8.59±5.16	8.13±2.60
Chol (mg/dL)	30	204.68±32.23	196.93±30.31	193.02±33.95	191.91±36.30
HDL-Chol (mg/dL)	30	64.08±14.25	62.19±13.00	53.86±14.07	55.44±16.96
Trig (mg/dL)	30	124.42±64.83	122.67±47.97	131.71±43.83	142.31±64.45
Na (mEq/L)	30	140.49±2.85	140.08±7.29	141.71±3.10	142.06±2.35
K (mEq/L)	30	4.06±0.47	5.94±13.27	4.10±0.37	4.16±0.47
Cl (mEq/L)	30	100.49±2.31	100.67±3.28	102.40±3.38	103.08±2.62
Ca (mg/dL)	30	9.33±0.40	11.28±12.47	9.40±0.36	9.39±0.27
P (mg/mL)	30	3.18±0.43	5.23±13.38	3.23±0.59	3.12±0.51
Mg (mEq/L)	30	1.99±0.19	3.97±13.56	2.10±0.17	2.11±0.11
Fe (µg/dL)	30	94.04±30.42	83.84±33.71	76.40±26.40	74.86±28.29
AST (u/L)	30	21.90±9.12	19.10±12.11	17.71±16.72	16.45±3.87
ALT (u/L)	30	15.00±7.47	13.66±13.11	12.75±6.99	13.81±4.79
γGT (u/L)	30	24.18±18.36	19.56±16.57	20.00±18.06	19.44±9.75
LDH (mU/mL)	30	199.38±37.39	191.36±45.27	184.48±41.62	175.06±29.58
CK (u/L)	30	105.75±104.39	91.45±57.21	67.00±42.55	90.68±79.22
Amy (u/L)	30	75.13±30.23	80.93±44.24	62.86±22.37	86.64±109.80
Lip (u/L)	30	39.40±31.21	44.39±61.05	30.00±14.22	30.11±8.87
Alb (mg/dL)	30	57.94±3.78	58.61±6.65	57.89±3.73	56.72±4.13
a-1GL (mg/dL)	30	4.04±0.55	6.00±13.27	4.15±0.65	4.12±0.53
α-2GL (mg/dL)	30	11.13±1.52	12.90±12.34	12.03±1.57	11.30±1.53
βGL (mg/dL)	30	11.52±1.40	13.61±12.14	11.39±1.22	12.46±1.67

**γGl (mg/dL)**	30	15.34±2.68	16.91±12.06	14.55±2.52	15.40±2.90
**ESR (mm/h)**	30	30.34±19.73	29.21±22.80	28.80±21.02	26.13±15.62
**FA (ng/mL)**	30	9.97±6.44	13.26±15.91	12.70±10.99	11.62±10.35
**VitB12 (pg/mL)**	30	684.52±869.45	787.80±1146.01	489.22±569.46	388.39±104.31
**PT (s)**	30	97.09±20.21	98.80±19.69	90.80±26.86	96.53±14.75
**INR ([])**	30	1.08±0.38	3.10±13.70	1.25±0.68	1.07±0.35
**PTT (s)**	30	29.76±3.30	31.60±10.07	31.15±4.11	30.46±2.99
**PTTr**	30	0.98±0.10	3.03±13.71	1.05±0.14	1.02±0.08
**Fibr (mg/mL)**	30	352.88±81.51	332.69±95.30	369.54±98.56	343.13±70.68
**Fer (ng/m)**	30	146.06±128.04	73.69±78.42	86.13±69.71	60.11±56.30
**WBC (10^3/µL)**	30	5.68±1.44	7.87±13.08	6.62±1.61	6.81±1.69
**RBC (10^3/µL)**	30	4.30±0.49	6.18±13.24	4.43±0.44	4.58±0.49
**Hb (g/dL)**	30	13.03±1.28	14.20±12.10	12.90±1.49	13.30±1.68
**Hct (%)**	30	39.02±3.67	38.70±9.04	39.13±3.75	40.00±4.20
**MCV (fl)**	30	91.03±4.98	88.86±4.90	88.31±4.54	87.49±6.23
**MCH (pg)**	30	30.40±1.94	30.81±9.76	29.08±1.92	29.06±2.51
**MCHC (g/dL)**	30	33.40±0.84	34.43±9.06	32.91±1.12	33.19±1.17
**Plt (10^3/µL)**	30	239.50±37.01	255.95±59.42	252.33±58.74	252.35±41.58
**Neu (10^3/µL)**	30	60.24±7.34	61.03±8.14	61.00±7.32	60.40±7.57
**Lym (10^3/µL)**	30	28.50±5.99	29.34±11.52	28.13±7.05	27.92±7.15
**Mon (10^3/µL)**	30	7.89±1.83	9.49±12.80	7.36±1.23	7.67±1.33
**Eos (10^3/µL)**	30	2.66±1.53	5.54±13.41	2.97±1.25	3.58±1.83
**Bas (10^3/µL)**	30	0.69±0.33	2.62±13.77	0.51±0.25	0.40±0.24
**Neu (%)**	30	3.46±1.08	5.59±13.37	4.06±1.15	4.16±1.33
**Lym (%)**	30	1.59±0.44	3.64±13.62	1.84±0.62	1.84±0.45
**Mon (%)**	30	0.43±0.11	2.48±13.79	0.48±0.12	0.51±0.13
**Eos (%)**	30	0.14±0.09	2.24±13.82	0.19±0.11	0.25±0.16
**Bas (%)**	30	0.03±0.01	2.07±13.85	0.03±0.01	0.02±0.01
**Palb (mg/dL)**	30	26.79±7.20	27.53±11.90	26.27±7.24	25.29±5.46
**GPx (u/L)**	30	47.82±18.25	51.46±18.50	49.22±20.04	47.91±15.36
**GR (µmol/L)**	30	6.34±3.10	8.25±13.59	5.99±2.82	5.90±2.05
**SOD (u/L)**	30	1361.98±546.01	1333.53±450.40	1443.41±530.67	1392.30±508.50
**TPAO**	30	1.18±0.35	1.10±0.35	1.03±0.28	1.07±0.31
**FRD (u/carr)**	30	353.12±89.86	357.07±101.97	305.24±85.04	293.18±73.07

Supplementary Table S2: results (p-value) of the statistical analysis comparing AD patients and control subjects. Analyte = Analyte acronyms; Mild = class vs. control subjects; Moderate = moderate class vs. control subjects; Severe = severe class vs. control subjects; Very severe = very severe class vs. control subjects. Statistically significant analytes are marked with "*".

Analyte	Mild	Moderate	Severe	Very severe
**Glu**	1.000	0.876	1.000	1.000
**Urea**	1.000	1.000	1.000	1.000
**Cre**	1.000	0.954	0.204	1.000
**LA***	0.013	0.620	0.111	0.019
**Bil**	1.000	1.000	0.534	1.000
**BA**	0.160	0.538	0.870	0.595
**Chol**	1.000	1.000	0.334	1.000
**HDL-Chol**	1.000	1.000	1.000	1.000
**Trig**	0.115	0.260	0.290	0.552
**Na**	1.000	1.000	1.000	0.149
**K**	1.000	1.000	1.000	1.000
**Cl ***	0.002	0.073	0.080	0.292
**Ca**	1.000	0.930	0.744	0.509
**P**	0.362	1.000	0.719	1.000
**Mg**	0.129	0.339	0.026	0.361
**Fe**	1.000	1.000	1.000	1.000
**AST ***	0.015	0.193	0.014	0.002
**ALT ***	0.008	0.035	0.014	0.404
**γGT**	1.000	1.000	0.340	1.000
**LDH**	0.167	0.678	0.068	0.857
**CK ***	0.313	0.701	0.016	0.037
**Amy**	1.000	1.000	1.000	1.000
**Lip**	1.000	1.000	1.000	1.000
**Alb**	0.140	0.899	1.000	0.992
**α-1Gl**	1.000	0.672	0.682	1.000
**α-2Gl**	0.299	1.000	0.025	0.538
**βGl**	0.333	0.590	1.000	1.000
**γGl**	0.683	0.487	1.000	1.000
**ESR**	0.815	1.000	1.000	1.000

**FA**	1.000	1.000	1.000	1.000
**VitB12**	0.074	0.075	0.246	0.333
**PT**	1.000	1.000	1.000	1.000
**INR**	1.000	1.000	1.000	1.000
**PTT ***	0.037	1.000	0.634	0.641
**PTTr**	1.000	1.000	1.000	1.000
**Fibr**	1.000	1.000	1.000	1.000
**Fer**	1.000	0.148	1.000	1.000
**WBC**	1.000	1.000	1.000	1.000
**RBC**	0.074	0.264	0.057	1.000
**Hb**	0.210	0.150	0.137	1.000
**Hct**	0.161	0.294	0.747	1.000
**MCV**	1.000	1.000	1.000	1.000
**MCH**	1.000	1.000	1.000	1.000
**MCHC**	1.000	0.313	0.614	0.099
**Plt**	1.000	1.000	1.000	1.000
**Nue**	0.692	0.309	0.887	0.992
**Lym**	0.460	0.286	1.000	1.000
**Mon**	1.000	1.000	1.000	1.000
**Eos**	0.348	0.375	1.000	1.000
**Bas**	1.000	1.000	1.000	1.000
**Neu%**	1.000	0.867	0.815	1.000
**Lym%**	0.169	0.164	1.000	1.000
**Mon%**	1.000	1.000	1.000	1.000
**Eos%**	0.559	0.398	0.950	1.000
**Bas%**	1.000	1.000	1.000	1.000
**Palb ***	0.003	0.004	0.007	0.003
**GPx**	1.000	1.000	0.210	1.000
**GR**	0.187	0.098	0.066	1.000
**SOD**	1.000	1.000	1.000	1.000
**TPAO**	1.000	0.709	0.820	0.644
**FRD**	1.000	1.000	1.000	1.000
